# 447. An Ordinal Scale Assessing SARS-CoV-2 Infected Patient Outcomes Using Electronic Health Records

**DOI:** 10.1093/ofid/ofab466.646

**Published:** 2021-12-04

**Authors:** Maryam Khodaverdi, Bradley S Price, Susan L Santangelo, Alfred (Jerrod) Anzalone, Wesley Kimble, J Zachary Porterfield, Michael T Vest, Sally L Hodder, Brian Hendricks, Clifford james Rosen, H TImothy Bunnell, Hamidreza Moradi

**Affiliations:** 1 WVCTSI, Morgantown, West Virginia; 2 West Virginia University, Morgantown, West Virginia; 3 Tufts University School of Medicine, Portland, Maine; 4 University of Nebraska Medical Center, Omaha, NE; 5 University of Kentucky College of Medicine, Lexington, Kentucky; 6 Christiana Care Healthcare System, Hockessin, Delaware; 7 West Virginia University School of Medicine, Morgantown, West Virginia; 8 Maine Medical Center Research Institute, scarborough, Maine; 9 Nemours Children’s Health System & University of Delaware, Wilmington, Delaware; 10 University of Mississippi Medical Center, JACKSON, Mississippi

## Abstract

**Background:**

A major challenge to identifying effective treatments for COVID-19 has been the conflicting results offered by small, often underpowered clinical trials. The World Health Organization (WHO) Ordinal Scale (OS) has been used to measure clinical improvement among clinical trial participants and has the benefit of measuring effect across the spectrum of clinical illness. We modified the WHO OS to enable assessment of COVID-19 patient outcomes using electronic health record (EHR) data.

**Methods:**

Employing the National COVID Cohort Collaborative (N3C) database of EHR data from 50 sites in the United States, we assessed patient outcomes, April 1,2020 to March 31, 2021, among those with a SARS-CoV-2 diagnosis, using the following modification of the WHO OS: 1=Outpatient, 3=Hospitalized, 5=Required Oxygen (any), 7=Mechanical Ventilation, 9=Organ Support (pressors; ECMO), 11=Death. OS is defined over 4 weeks beginning at first diagnosis and recalculated each week using the patient’s maximum OS value in the corresponding 7-day period. Modified OS distributions were compared across time using a Pearson Chi-Squared test.

**Results:**

The study sample included 1,446,831 patients, 54.7% women, 14.7% Black, 14.6% Hispanic/Latinx. Pearson Chi-Sq P< 0.0001 was obtained comparing the distribution of 2^nd^ Quarter 2020 OS with the distribution of later time points for Week 4.

Table 1. OS at week 1 and 4 by quarter

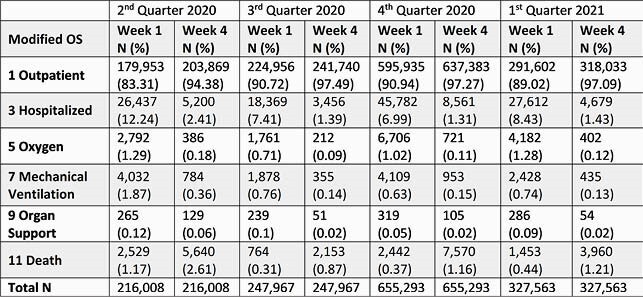

The study sample included 1,446,831 patients, 54.7% women, 14.7% Black, 14.6% Hispanic/Latinx. Pearson Chi-Sq P< 0.0001 was obtained comparing the distribution of 2nd Quarter 2020 OS with the distribution of later time points for Week 4.

**Conclusion:**

All Week 4 OS distributions significantly improved from the initial period (April-June 2020) compared with subsequent months, suggesting improved management. Further work is needed to determine which elements of care are driving the improved outcomes. Time series analyses must be included when assessing impact of therapeutic modalities across the COVID pandemic time frame.

**Disclosures:**

**Sally L. Hodder, M.D.**, **Gilead** (Advisor or Review Panel member)**Merck** (Grant/Research Support, Advisor or Review Panel member)**Viiv Healthcare** (Grant/Research Support, Advisor or Review Panel member)

